# Neuroinflammation and Dyskinesia: A Possible Causative Relationship?

**DOI:** 10.3390/brainsci14050514

**Published:** 2024-05-20

**Authors:** Antonella Cardinale, Antonio de Iure, Barbara Picconi

**Affiliations:** 1 Experimental Neurophysiology Laboratory, IRCCS San Raffaele Roma, 00166 Rome, Italy; antonella.cardinale@sanraffaele.it (A.C.); antonio.deiure@uniroma5.it (A.d.I.); 2 Department of Human Sciences and Quality of Life Promotion, Università Telematica San Raffaele, 00166 Rome, Italy

**Keywords:** Parkinson’s disease, levodopa (L-DOPA), L-DOPA-induced dyskinesias (LIDs), non-neuronal mechanism, neuroinflammation

## Abstract

Levodopa (L-DOPA) treatment represents the gold standard therapy for Parkinson’s disease (PD) patients. L-DOPA therapy shows many side effects, among them, L-DOPA-induced dyskinesias (LIDs) remain the most problematic. Several are the mechanisms underlying these processes: abnormal corticostriatal neurotransmission, pre- and post-synaptic neuronal events, changes in gene expression, and altered plasticity. In recent years, researchers have also suggested non-neuronal mechanisms as a possible cause for LIDs. We reviewed recent clinical and pre-clinical studies on neuroinflammation contribution to LIDs. Microglia and astrocytes seem to play a strategic role in LIDs phenomenon. In particular, their inflammatory response affects neuron-glia communication, synaptic activity and neuroplasticity, contributing to LIDs development. Finally, we describe possible new therapeutic interventions for dyskinesia prevention targeting glia cells.

## 1. Introduction

Parkinson’s disease (PD) is the second most common neurodegenerative disease, characterized by progressive dopaminergic neuron loss and α-synuclein aggregated clusters, which affect movement circuits [1,2,3]. In recent years the prevalence and incidence of PD has been increasing significantly, leading to an increase in disability and therefore increasing costs for national health systems [4,5]. PD is a multifactorial disease caused both by genetic modification and different environmental factors [6]. Moreover, PD is a disorder characterized by a combination of motor symptoms (including tremor, rigidity, gait abnormality, and bradykinesia) and non-motor symptoms [7].

The dopamine (DA) precursor l-3,4-dihydroxyphenylalanine (L-DOPA) remains the most successful treatment, prescribed in conjunction with carbidopa [7,8]. Unfortunately, the lifelong treatment needed involves several adverse reactions and side effects, among which levodopa-induced dyskinesias (LIDs) are the most urgent problem to be solved. LIDs are characterized by abnormal involuntary movements, such as stereotypic, choreiform, and throwing movements, as well as dystonia [9,10] involving the head, neck, trunk, and limbs [8,11]. About 80% of patients suffer from this inconvenience, which worsens their disability and their quality of life [8], after an average of 6.5 years of L-DOPA treatment [9,10]. However, in rodent PD models, exemplars did not manifest LID after 6-OHDA lesion and L-DOPA treatment [12], and about 6–22% of PD patients did not ever suffer LID [9,13,14,15,16,17]. 

Thus far, research in dopamine-lesioned animals has discovered several mechanisms underlying these processes, such as abnormal corticostriatal neurotransmission, pre- and post-synaptic neuronal events, changes in gene expression, altered synaptic plasticity [18], and dendritic spine reshaping [19]. 

In recent years, researchers have also suggested non-neuronal mechanisms as a possible cause of LIDs [20]. Here, we review recent clinical and pre-clinical studies on neuroinflammation contribution to LIDs. Microglia and astrocytes seem to play a crucial role in LIDs phenomenon. In particular, their inflammatory response affects neuron–glia communication, synaptic activity, and neuroplasticity, contributing to LID development. Finally, we describe possible new therapeutic interventions for dyskinesia prevention targeting glia cells.

## 2. Pathophysiology of LIDs

### 2.1. General Features

**Figure 1:**
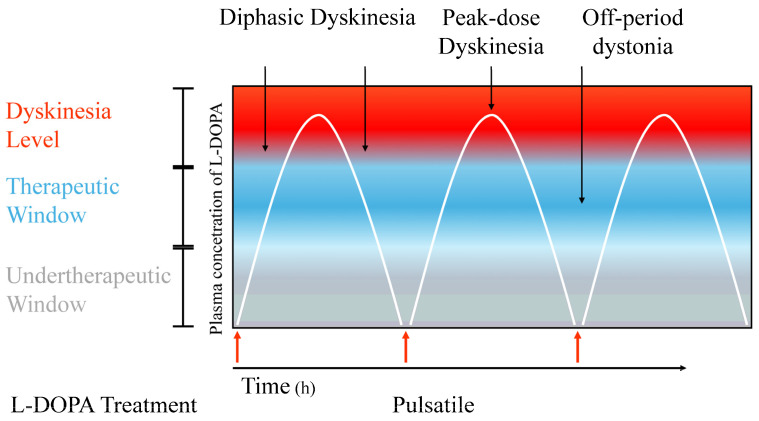
Different types of levodopa-induced dyskinesias. The figure accurately describes the different types of levodopa-induced dyskinesias. Diphasic dyskinesias occur early in the effect of levodopa treatment, before the peak of clinical benefit on motor symptoms is reached, and may recur as the drug effect wanes until it disappears. Peak dyskinesias coincide with the full antiparkinsonian benefit of levodopa during the “on” period, whereas “off” period dyskinesias occur when levodopa is no longer effective.

LIDs are characterized as three different types: “peak-dose dyskinesia or improvement-dyskinesia-improvement” (IDI) dyskinesia, diphasic dyskinesia or “dyskinesia-improvement dyskinesia” (DID) dyskinesia, and early-morning dystonia or off-period dyskinesia (Figure 1) [8,21]. The most frequent dyskinesia kind is peak-dose dyskinesia IDI dyskinesia (about 75–80% of patients experienced it), occurring during the so-called “on” time when the L-DOPA blood levels reached the peak [8,21,22].

Dopaminergic system and motor circuits depend on a correct equilibrium between direct and indirect pathways through DA binding to dopaminergic D1 or D2 receptors, respectively [23]. The alteration of direct and indirect pathways leads to a PD condition in which overstimulation of the internal globus pallidus (GPi) occurs with the consequent inhibition of the motor thalamus and the limitation of the activity of the corresponding motor area [24,25]. As described above, L-DOPA represents the main treatment for PD patients, which has different consequences depending on disease stage (early or late stages). In particular, during the earlier stages of PD when dopaminergic denervation is lower, DA, obtained from L-DOPA oral administration, is stored in the presynaptic vesicles, maintaining stable levels in these patients [26]. In contrast, when the majority of dopaminergic terminals on striatal brain region are lost, dopaminergic transporters are not able to store exogenous DA, resulting in the overstimulation of receptors due the high synaptic DA levels [27]. In fact, LID phenomena are associated with plasma L-DOPA concentration fluctuations [28] due to the short half-life of this drug [29,30].

### 2.2. Principal Mechanisms Involved

Many other factors are involved in the LID mechanisms [8,20]. Indeed, L-DOPA is usually processed by the enzyme aromatic L-amino acid decarboxylase (AADC), and decarboxylated to DA in the nigrostriatal dopaminergic fibers [31,32]. However, exogenous L-DOPA metabolism can occur both in serotonergic and noradrenergic terminals, due to their expression of the enzyme aromatic L-amino acid decarboxylase (AADC) [26,33]. Striatal synaptic plasticity is regulated by the interaction between dopaminergic and serotonergic systems [34]. Moreover, as described previously, the serotoninergic system contributes to L-DOPA metabolism. Unfortunately, serotoninergic 5-HT fibers do not show the capacity to regulate DA release due to the deficiency of D2 autoreceptors and DA transporters [33]. Thus, the effect is the fluctuations of DA levels in the synaptic cleft and an aberrant stimulation of striatal projection neurons (SPNs) [35,36,37,38]. A similar phenomenon happens in the noradrenergic terminals with the synthesis of L-DOPA into DA thanks to the AADC enzyme, also causing variable DA levels in the striatum [39].

Additionally, during the period of L-DOPA intake, D1 receptors are overstimulated with overactivity of the direct pathway [40,41] and strong activation of the cyclic adenosine monophosphate (cAMP) signaling pathway [18,42,43]. This activation in turn involves the stimulation of other downstream factors, such as cAMP-dependent protein kinase A (PKA), the dopamine- and cAMP-regulated protein 32 kDa (DARPP-32), the extracellular signal-regulated kinases (ERK), and the mammalian target of rapamycin (mTOR) pathways [33,42,43,44]. Moreover, phosphodiesterase 10 (PDE10) is involved in DA signaling, controlling PKA/DARPP-32 and cAMP signaling cascades [45]. PDE10A levels in the caudate putamen striatal region of PD patients are reduced, confirming their possible role into LID symptoms’ onset [46].

Additionally, increased glutamatergic neurotransmission, from the cortex to the striatum, occurs after dopaminergic cell loss and DA replacement therapy. Indeed, the NMDA (n-methyl-d-aspartate) and AMPA (α-amino-3-hydroxy-5-methyl-4-isoxazole propionic acid) receptors undergo cellular changes such as localization, post-translational modifications and changes to the subunit structure, contributing to LIDs’ progress [47,48,49,50,51,52]. NMDAR is an ionotropic glutamate receptor that is specifically involved in synaptic and neuroinflammatory processes. As a voltage- and ligand-gated cation channel, NMDAR presents a composition with different subunits. GluN1 and GluN2 are the most available isoforms, although we can also find GluN2A, GluN2B, GluN2C, and GluN2D [10,53,54,55]. These subunits will affect channel functions through their composition, distribution, and phosphorylation. Experience-generated neural activity can alter brain function by modifying synaptic transmission, known as synaptic plasticity. Altered synaptic plasticity is frequently observed in neurodegenerative diseases and can contribute to neurophysiological disorders. Synaptic plasticity can exist in several forms, both short- and long-term, and can have a depressor or potentiating effect. Synaptic plasticity is the mechanism that allows the realization of learning and memory processes. It is closely dependent on glutamatergic excitatory transmission through the modulation of NMDA receptors, which are controlled by dopaminergic pathways via binding to D1 and D2 receptors. The forms of synaptic plasticity that will be evaluated here are LTP (long-term potentiation) and LTD (long-term depression), which can be directly altered by inflammatory processes [1,2]. Indeed, synaptic plasticity and the induction of LTP and LTD are controlled by the phosphorylation grade of NMDAR isoforms, which in turn trigger learning and memory processes [10,18,56,57]. Motor signals, which reach the basal ganglia from the cortex, depend on the bidirectional control of synaptic plasticity by LTP and LTD. In the PD condition, even if the cortex sends information to the basal ganglia, the striatum loses the capacity to export appropriate signals; this phenomenon is amplified during LID onset [10,58]. In LID pathology, NMDAR can induce an overactivation of LTP which, with the contemporary lack of LTD, leads to redundant movements due to the abnormal motor information, as demonstrated in rat models [10]. 

Ultimately, it seems that not only drug factors are involved in LID onset but also PD patients’ own characteristics, such as oxidative-inflammatory marker genetic panels [59,60,61]. 

## 3. Neuroinflammation and Its Role in LID Development

As recently reviewed by our group, neuroinflammation contributes actively to the pathophysiology of PD [1,2,62]. As noted above, PD is a multifactorial disorder, characterized by synaptic damage, dopaminergic neuronal death, misfolded α-syn aggregation, and an altered immune response [1,2]. In summary, gradual dopaminergic cell death prompts the alteration of long-term potentiation (LTP) modifying the excitability of SPNs. While the degeneration continues, other processes affect it, producing a vicious cycle. Among the symptoms is the well-known inflammatory response present in the brain of PD patients by means of microglia and astrocyte reactions, the gene expression of pro-inflammatory species, and the activation of adaptive immune factors from the periphery [1,2,63,64,65,66,67]. Indeed, cytokines, when secreted in excess by microglia, are involved in LID development, affecting corticostriatal synaptic plasticity and glutamatergic transmission [68,69,70]. Interestingly, the activation of inflammation already occurs when dopaminergic terminal death and plasticity alteration arise, but long before the death of dopaminergic neurons [1,2,71,72]. Moreover, as previously described by our group, extracellular α-syn, acting as damaged-associated molecular patterns (DAMPs), elicits an inflammatory cascade inducing chemokine and cytokine production from microglia [1,2]. Indeed, a number of studies have indicated that the dorsal striatum of dyskinetic rats has a higher rate of glia cell reactions [73]. 

Thus, neuroinflammation represents a well-known altered process in PD pathology. Numerous pre-clinical studies have identified the quality and quantity changes of microglial and astrocytes’ population with releasing of immune system agents such as interleukin-1β (IL-1β), inducible nitric oxide (NO), nitric oxide synthase (iNOS), tumor necrosis factor-a (TNF-a), cyclooxygenase-2 (COX-2) enzyme, and chemokines. Indeed, treatment with anti-inflammatory drugs, such as ibuprofen (a non-selective COX inhibitor) or glucocorticoid corticosterone [74], seems to be able to fight the immune response and to decrease dyskinesia symptoms in PD animal models, significantly enhancing the efficiency and tolerance of L-DOPA treatment [73,75,76,77]. 

**Figure 2:**
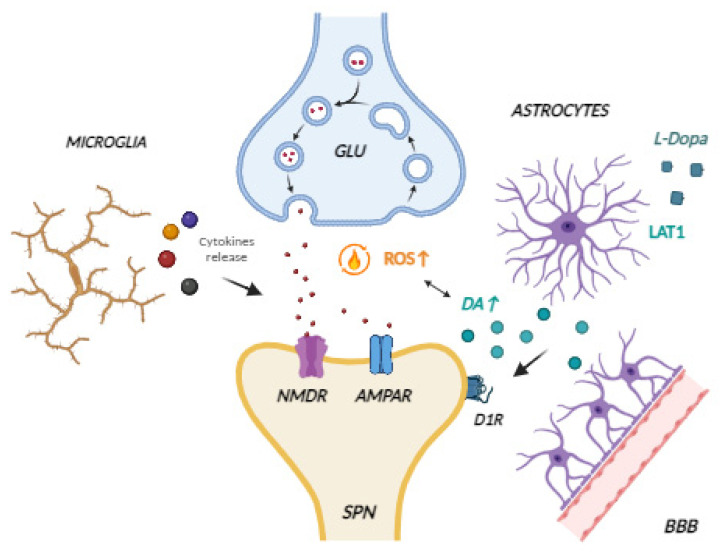
The involvement of neuroinflammation in LID. LIDs are abnormal involuntary movements due to the daily treatment with L-DOPA. Several are the mechanisms underlying these processes: abnormal corticostriatal neurotransmission, pre- and post-synaptic neuronal events, changes in gene expression, and altered plasticity. In recent years, researchers have also suggested non-neuronal mechanisms as a possible cause for LID. Indeed, cytokines, when secreted in excess by microglia, are involved in LID development affecting corticostriatal synaptic plasticity and glutamatergic transmission [68,69,70]. Among the causes of the inflammatory response are the increased oxidation due to excessive DA concentration, and astroglia transport and distribution of L-DOPA from the blood to brain tissues. Indeed, astrocytes take up L-DOPA through an amino acid transporter, named L-type amino acid transporter 1 or sodium-independent neutral amino acid transporter (LAT1). Astrocytes appear to act as a DA storage facility and to release it only based on its extracellular concentration. Additionally, astroglia presents as a DA transporter, able to internalize DA derived from L-DOPA metabolism. Indeed, astrocytes also possess monoamine oxidase (MAO-B) and catechol-O-methyltransferase (COMT) and thus participate in L-DOPA metabolism [68,82,83,84,85] (Figure created in BioRender.com). GLU: glutamatergic neuron and glutamate; SPNs: striatal projection neurons.

In the past decade, studies have focused on a possible role for inflammatory outbreaks in the LID pathological mechanism. The inflammatory response caused by daily treatment with L-DOPA and LIDs can be attributed to a variety of reasons, which are listed below (Figure 2).

First of all, neuroinflammation seems to affect NMDAR function and expression, even during the LID phenomenon. Importantly, cytokines (TNF-α, IL-1β, NO, iNOS) and chemokines control the release of glutamic acid (Glu) from presynaptic neurons and the expression of Glu receptors in postsynaptic neurons [10,78,79]. As noted above in this review, GluN1 and GluN2 are the most expressed subunits of the NMDAR, whose phosphorylation contributes to dysregulation of synaptic plasticity and the trigger of LIDs.

Second, astroglia seems to transport and distribute L-DOPA from the blood to brain tissues. As is well-known, the blood–brain barrier (BBB) is composed of astrocytic end-feet [20,68,80,81]. Indeed, astrocytes take up L-DOPA through an amino acid transporter, named L-type amino acid transporter 1 or sodium-independent neutral amino acid transporter (LAT1) [82]. Astrocytes appear to act as a DA storage facility and to release it only based on its extracellular concentration [68,83]. Additionally, astroglia presents as a DA transporter, able to internalize DA derived from L-DOPA metabolism [84]. Indeed, astrocytes also possess monoamine oxidase (MAO-B) and catechol-*O*-methyltransferase (COMT) and thus participate in L-DOPA metabolism [85].

Accumulating research evidence has demonstrated the inflammatory response contribution to LIDs by the contribution of microglia and soluble pro-inflammatory cytokines (TNF-α, Il-1β, iNOS) [20,86]. As demonstrated by Mulas and co-workers in 2016, a dyskinetic L-DOPA treatment also induced microglial reactivity with increased TNF-α expression in contrast to a different L-DOPA administration, defined non-dyskinetic, through continuous subcutaneous infusion [20,87]. Recently, Morissette and collaborators have demonstrated the correlation between increased inflammatory markers and LID scores in dyskinetic monkeys compared to that of vehicle-treated MPTP monkeys, not only in the basal ganglia but also in other downstream basal ganglia nuclei including the GPe and GPi. Moreover, treatment with MPEP (a metabotropic glutamate receptor 5 antagonist) reduced both the development of LID in de novo MPTP-lesioned monkeys and the inflammatory reaction measured by means of IBA1, CD68, and GFAP markers [88]. As is well known, PD is closely associated with advancing age and at the same time also affects the LID onset. In particular, there is an increased risk of developing LIDs when juvenile PD onset occurs [89,90,91]. A recent work has demonstrated this issue, by conducting experiments in both adult (3 months) and juvenile (18 month) male Fischer rats, bearing unilateral 6-hydroxydopamine (6-OHDA)-lesions of the medial forebrain bundle. The animals were treated acutely with a vehicle or L-DOPA (6 mg/kg) [89]. The research group has investigated the relationship between LIDs and aging-derived neuroinflammation. The outcome of this study showed the increase of IL-1β gene expression in the striatum ipsilateral and lesions only in the group of 18-month-old rats, confirming a previous study in mice [89,92]. Indeed, an IL-1β receptor antagonist infusion in the striatum effectively decreased LIDs in the 6-OHDA model [75]. Recently, a study in a rodent model of PD, chronically treated with L-DOPA to provoke abnormal involuntary movements (AIMs), revealed via transcriptomic analyses the involvement of the following factors as key molecular mediators of LID advancement: transforming growth factor beta type 1 (TGFβ1), interleukin 1 beta (IL1β), and tumor necrosis factor alpha (TNFα) cytokines as key mediators of chronic inflammation [93]. Additionally, TGFβ1, IL-1-β, and TNFα alterations were found in cerebrospinal fluid (CSF) and cerebral tissues of PD patients [93].

Indeed, TNF-α represents another proinflammatory cytokine that affects dyskinesia pathophysiologic machinery [94,95]. In particular, TNF-α is an effective activator of resting microglia and a modulator of LTP by the modulation of AMPA glutamate receptor subunit 1 (GLUR1) expression, which is implicated in LIDs [96,97,98,99]. Furthermore, TNF-α controls neuronal excitability and synaptic plasticity, through TNF receptor 1 and 2 (TNR1 and TNFR2) [68,96,100,101]. In addition, TNF-α affects the synaptic activity altering AMPA receptor [68,100]. The inhibition of TNF-α through thalidomide (TLD) and its derivative 3,6’-dithiothalidomide (DTT) in the 6-OHDA rat model of PD attenuated LID scores via an anti-angiogenic activity in basal ganglia and an overexpression of GLUR1 [102,103,104,105,106,107,108,109].

Another important player in the molecular inflammatory mechanisms is represented by IFN-γ, a pro-inflammatory cytokine involved in the iNOS transcription [110]. Moreover, IFN-γ stimulates glial cells, and PD patients present augmented levels in the brain and in the plasma; thus, this cytokine could have a key role in the PD pathology [111]. Investigators showed astrocyte and iNOS reactivity in IFN-γ/KO parkinsonian mice without affecting the dopaminergic cells death or LID onset, suggesting that neuroinflammation could be arising by L-DOPA in a different pathway, aside from IFN-γ signaling [111].

Another key role in the neuroinflammation could be that of increased oxidation due to excessive concentration and metabolism of DA, following the long-term use of the L-DOPA drug. Interestingly, L-DOPA, as the chemical precursor of dopamine, prompts the production of free radicals, which worsens the oxidative damage and alteration characteristic of Parkinson’s disease pathology. Indeed, some authors support the idea that some L-DOPA quantity is converted into dopamine, despite the medical prescription of DOPA-decarboxylase inhibitors. The increment of free radicals and the consequent oxidative stress could be able to induce the occurrence of dyskinesia pathology. In addition, specific experimental studies have drawn attention to the potential effects of synthetic anti-oxidants on the amelioration of hyperkinetic movements in LID animal models. In the context of inflammatory oxidative pathophysiology of PD, Sarkar and his collaborators have conducted a study on PD patients presenting or not with dyskinesia, compared to healthy-matched people [112]. They found a different oxidative profile and inflammatory response in PD with or without LID. In particular, reduction of antioxidant activity, and TOLLIP (toll interacting protein) and IL-1β upregulation were found in LID patients compared to controls. TOLLIP is an inhibitory adaptor protein of the TRL pathway, involved in the endo-lysosomal degradation of IL-1R, and its overexpression inhibits inflammatory processes. Other investigations have highlighted the involvement of the nitric oxide (NO) signaling pathway. NO is a neurotransmitter synthesized from its precursor L-arginine. Furthermore, NO seems to be involved in inflammatory events of PD. Indeed, neuronal NOS (nNOS) mRna [113], nNOS, and inducible NOS (iNOS) protein [76,114] presented an increased concentration level in L-DOPA-induced dyskinesia model rats. Of note, the nNOS inhibitors were effective in preventing dyskinesia manifestation and COX2 increased expression, which is also involved in LID development [112]. 

## 4. Therapeutic Interventions

As previously mentioned, LIDs represent the most frequent adverse effect of daily treatment with L-DOPA. As is known, PD prevalence is increasing due to many factors and in particular the aging world population. Furthermore, all these lead to an increase in socioeconomic costs and deterioration in the quality of life of PD-affected people, impairing daily activities (such as eating and drinking) and enhancing anxiety and depression as well as the risk of falling [115,116,117,118,119]. Currently, the only treatment approved and the most efficient drug for the management of LIDs is Amantadine, a low-affinity non-competitive NMDA receptor antagonist, as reported by several clinical studies [7,11,115,120]. Amantadine is available in two formulations, only one approved, and one used off label [121]. The latter formulation of Amantadine was approved by the FDA in 2017 and presents an extended release [115]. However, Amantadine presents side effects such as confusion and hallucinations [122]. Indeed, Amantadine acts by reducing the inflammatory response induced by microglia [93,123]. This supports the role of neuroinflammation and its use as a therapeutic target in the fight against LIDs. In this way, corticosterone, a hormone with potent immunomodulatory properties, seemed to reduce LIDs development when administered prior to L-DOPA treatment [75]. As described above, treatment with MPEP (a metabotropic glutamate receptor 5 antagonist) reduced the onset of LIDs in de novo MPTP-lesioned monkeys through decrease of inflammatory reaction by means of IBA1, GFAP, and CD68 in the basal ganglia without effects in the nucleus accumbens and motor cortex M1 [88]. An alternative treatment for LIDs in PD could be the use of doxycycline (doxy), a semisynthetic tetracycline antibiotic with anti-inflammatory characteristics able to pass the BBB [124]. Doxycycline triggered the reduction of LIDs in L-DOPA-treated dyskinetic mice by decreasing Fos-B and COX-2 expression and lowering PGE_2_, TNF-α, and IL-1β levels in the dorsolateral striatum [124,125].

Among anti-inflammatory compounds, there is methylene blue (MB), a non-selective inhibitor of the soluble enzyme guanylyl cyclase (sGC) involved in the signaling pathway for nitric oxide (NO) transmission [98,126,127]. Bariotto-Dos-Santos and collaborators have demonstrated that co-administration of MB with L-DOPA reduced the risk of LID development, probably due to its anti-inflammatory properties, leading to a decrease in microglia reaction and expressions of pro-inflammatory cytokines [125,128,129,130].

From the point of view of prevention, in this review, we should mention nutraceutical products tested in animal models to support pharmacological therapy. Among them, resveratrol (trans-3, 4, 5-trihydroxystilbene, RES) seems to have many beneficial effects. In fact, it has anti-oxidative, anti-aging, anti-inflammatory, anti-cancer, and anti-microbial properties, and the ability to cross the blood–brain barrier (BBB), acting on the central nervous system. Moreover, research on PD animal models has demonstrated that RES could rescue Da neurons and reduce L-DOPA side effect, such as severity of dyskinesia, probably due to its anti-inflammatory characteristics [131]. Ultimately, preclinical studies have shown that cholecalciferol (VD3) treatment ameliorates motor impairments and diminished IL-1β and CD11b inflammatory expression [132]. Indeed, VD3 deficiency has been found in various neurological disorders [133], including PD. VD3 is a steroid involved in gene expression, whose receptor is largely concentrated in striatum [134]. Moreover, VD3 demonstrated anti-dyskinetic properties by mitigation of dyskinetic abnormal involuntary movements (AIMs), due to its ability to modulate the microglia reaction, generation of ROS, inflammation, and apoptotic pathways, not involving dopaminergic modulation. All treatments are noted in Table 1.

The current presented review supports the urgent need for further studies to deepen understanding of the inflammatory mechanism in LIDs. Drug repurposing could be a crucial strategy to investigate various drugs available for LID treatment.

## 5. Conclusions

Currently, treatment with L-DOPA remains the gold standard therapy for PD patients due to its efficacy. However, it is important to note that this treatment can lead to disabling side effects, including motor and cognitive complications, which may contribute to the onset of LID. This article reviews recent literature that highlights the correlation between PD and neuroinflammation and how inflammatory processes are closely linked to the onset of LID. It is clear that a potential area for research is the expansion of pharmacological methods to delay the onset of LID or limit its impact, particularly in relation to neuroinflammation. The multifactorial nature of PD requires the evaluation of new pharmacological strategies, bearing in mind the potential risk of developing levodopa-induced dyskinesias (LID). Therefore, we suggest that the reduction of side effects due to the activation of inflammatory processes during the progress of PD should not be neglected, as they are likely to be involved both in neurodegeneration itself and in the development of LIDs. As such, neuroinflammation represents a new field of research and could be an excellent therapy target both to slow down the progression of PD and to reduce LIDs, bettering the quality of life of PD patients.

## 6. Limitations of the Studies

In this review, we wanted to analyze the literature on the non-neuronal mechanisms involved in the dyskinesia onset. In this context, we also analyzed the possible drug targets or treatments that could be used. However, there are several limitations in the use of these novel drugs for several reasons:

- Could have serious adverse reactions;
- Could reduce the effectiveness of L-dopa;
- Have excellent results in pre-clinical practice but no evidence in medical use due to the lack of clinical trials.

Therefore, in particular, it is urgent to link the pre-clinical studies to human trials on anti-inflammatory protocols to assess evidence of beneficial effects for PD patients and to associate them with the LID pharmacological treatments. Obviously, this urgency must also be applied to the other non-neuronal mechanisms involved to amplify the effectiveness of treatments.

## Figures and Tables

**Table 1:** Anti-inflammatory drug treatments in LID.

Drug Name	Characteristics	References
Amantadine	As a mild glutamate receptor antagonist, it is used to treat Parkinson’s disease (PD), boosting dopamine and preventing its reuptake	[7,11,115,120]
Corticosterone	Hormone with potent immunomodulatory properties	[75]
Ibuprofen	A non-selective COX inhibitor	[74]
MPEP	Metabotropic glutamate receptor 5 antagonist	[88]
Doxycycline	A semisynthetic tetracycline antibiotic	[124]
Methylene blue (MB)	A non-selective inhibitor of the soluble enzyme guanylyl cyclase (sGC)	[98,125,126,127,128,129,130]
Resveratrol (trans-3, 4, 5-trihydroxystilbene, RES)	Class of plant micronutrients called polyphenols	[131]
Cholecalciferol (VD3)	Vitamin	[132,133,134]

## Abbreviations

AADC	Aromatic L-Amino Acid Decarboxylase
AIMs	Abnormal Involuntary Movements
BBB	Blood–Brain Barrier
cAMP	Cyclic Adenosine Monophosphate
CD68	Cluster of Differentiation 68
CSF	Cerebrospinal Fluid
COMT	Catechol-*O*-Methyltransferase
DA	Dopamine
DARPP-32	Dopamine and Camp-Regulated Protein of 32 kDa
DAT	Dopamine Transporter
DID	Dyskinesia-Improvement Dyskinesia
ERK	Extracellular Signal-Regulated Kinases
IBA1	Ionized Calcium Binding Adaptor Molecule 1
IDI	Improvement-Dyskinesia-Improvement
GFAP	Glial Fibrillary Acidic Protein
GPi	Internal Globus Pallidus
Il-1β	*Interleukin-1* beta
iNOS	Inducible NO Synthase
IFN-γ	Interferon Gamma
LAT1	L-Type Amino Acid Transporter 1
LID	L-DOPA-Induced Dyskinesia
L-DOPA	l-3,4-Dihydroxyphenylalanine Levodopa
LTD	Long-Term Depression
LTP	Long-Term Potentiation
MAO-B	Monoamine Oxidase
MPEP	Metabotropic Glutamate Receptor 5 Antagonist
MPTP	1-Metil 4-Fenil 1,2,3,6-Tetraidro-Piridina
mTOR	Mammalian Target of Rapamycin
SPNs	Striatal Projection Neurons
PDE10	Phosphodiesterase 10
PKA	cAMP-dependent Protein Kinase A
PD	Parkinson’s Disease
TGFβ1	Transforming Growth Factor beta type 1
TNF-α	Tumor Necrosis Factor
TNFR2	TNF Receptor 2
TNR1	TNF Receptor 1
